# Thyroid hormone does not induce maturation of embryonic chicken cardiomyocytes in vitro

**DOI:** 10.14814/phy2.12182

**Published:** 2014-12-11

**Authors:** Ann‐Charlotte B. Svensson Holm, Isa Lindgren, Hanna Österman, Jordi Altimiras

**Affiliations:** 1 IFM Biology, AVIAN Behaviour Genomics and Physiology Group, Linköping University, Linköping, Sweden

**Keywords:** Heart growth, hyperplasia, hypertrophy, insulin‐like growth factor‐1, thyroid hormone

## Abstract

Fetal cardiac growth in mammalian models occurs primarily by cell proliferation (hyperplasia). However, most cardiomyocytes lose the ability to proliferate close to term and heart growth continues by increasing cell size (hypertrophy). In mammals, the thyroid hormone triiodothyronine (T_3_) is an important driver of this process. Chicken cardiomyocytes, however, keep their proliferating ability long after hatching but little information is available on the mechanisms controlling cell growth and myocyte maturation in the chicken heart. Our aim was to study the role of T_3_ on proliferation and differentiation of embryonic chicken cardiomyocytes (ECCM), enzymatically isolated from 19‐day‐old embryos and to compare the effects to those of insulin‐like growth factor‐1 (IGF‐1) and phenylephrine (PE). Hyperplasia was measured using a proliferation assay (MTS) and hypertrophy/multinucleation was analyzed morphologically by phalloidin staining of F‐actin and nuclear staining with DAPI. We show that IGF‐1 induces a significant increase in ECCM proliferation (30%) which is absent with T_3_ and PE. PE induced both hypertrophy (61%) and multinucleation (41%) but IGF‐1 or T_3_ did not. In conclusion, we show that T_3_ does not induce maturation or proliferation of cardiomyocytes, while IGF‐1 induces cardiomyocyte proliferation and PE induces maturation of cardiomyocytes.

## Abstract (precis)

Our main findings in the study show that T_3_ does not affect proliferation or maturation of embryonic chicken cardiomyocytes (ECCMs). Furthermore, phenylephrine induces maturation of ECCMs and IGF‐1 act as a pro‐proliferative mediator.

## Introduction

Cardiac growth during fetal mammalian development takes place by cardiomyocyte proliferation (hyperplasia) (Smolich et al. 1989; Austin et al. 1995; Mayhew et al. 1997). Once cardiomyocytes undergo differentiation, they become binucleated and the heart can only grow by cell enlargement (hypertrophy) (Thornburg et al. 2011). After binucleation, the cells rarely enter the cell cycle again (Clubb and Bishop 1984) and are regarded as postproliferative and terminally differentiated.

The timing of this transition varies largely between species and it seems to be correlated with the maturation of the thyroid gland and the thyroid hormonal axis. In species where the thyroid is functional already during the last trimester of gestation such as humans, sheep, and pigs (Polk 1995), the rise in triiodothyronine (T_3_) concentration is correlated with the onset of cardiac binucleation (Barbera et al. 2000; Burrell et al. 2003). Indeed, isolated fetal sheep cardiomyocytes become binucleated upon T_3_ stimulation (Chattergoon et al. 2007). In altricial species showing postnatal maturation of the thyroid hormonal axis such as mice and rats, cardiomyocyte binucleation occurs at 1–2 weeks of age (Clubb and Bishop 1984; Cluzeaut and Maurer‐Schultze 1986; Soonpaa et al. 1996), coincident with the time at which heart regeneration is no longer possible in the species (Porrello et al. 2011).

In this context, the chicken presents an interesting conundrum because thyroid axis maturation occurs already during fetal development but cardiomyocyte proliferation continues at least until 6 weeks post hatching (Li et al. 1997). Sharp rises in T_3_ prior to hatching have been reported in chickens (Thommes and Hylka 1977; Christensen et al. 1995) with a pattern very similar to that in sheep and humans (Polk 1995). In sheep, the T_3_ surge prior to birth is driven by glucocorticoids during natural parturition (Klein et al. 1978) or when exogenous cortisol is administered to induce parturition (Thomas et al. 1978), which is similar to the corticosterone induced T_3_ surge also seen in chickens (Decuypere et al. 1983; Kuhn et al. 1984).

Unlike the sheep, however, where 70% of the cardiomyocytes are binucleated at term (Jonker et al. 2007), binucleation is essentially absent from chicken cardiomyocytes at the time of hatching (Li et al. 1997). Proliferation is suppressed toward term in the chicken (Jeter and Cameron 1971) but T_3_ in itself does not hinder proliferation although it reduces the proliferative activity of basic fibroblast growth factor (Kardami 1990). Proliferation increases immediately post hatching (Jeter and Cameron 1971) and it is maintained at least until 6 weeks of age, where 44% of the cardiomyocytes are still mononucleated (Li et al. 1997).

Therefore, we designed our study to explore the role of T_3_ on cardiomyocyte proliferation and differentiation in chickens and we compared the effects to those of IGF‐1, a stimulus of cell proliferation (Kardami 1990; Resnicoff et al. 1993; Cao et al. 2003; Sundgren et al. 2003b) and phenylephrine, a hypertrophic stimulus (Yue et al. 2000).

## Materials and Methods

### Chemicals

Chemicals used: Bodipy phallacidin (Molecular Probes, Eugene, OR); Dulbecco's Modified Eagle Medium, nonessential amino acids, sodium pyruvate, penicillin and streptomycin (PEST), fetal bovine serum (FBS), and Trypsin‐EDTA (Gibco, Paisley, Scotland); TRI‐reagent, monoclonal anti‐*α*‐actin antibody, insulin‐like growth factor‐1 (IGF‐1), 3, 3',5‐Triiodo‐L‐thyronine sodium salt (T_3_), anti‐*α*‐actin, bovine serum albumin (BSA), protease, and saponin (Sigma Chemical Co., St. Louis, MO); CellTiter 96^®^ Aqueous One solution cell proliferation (MTS) assay (Promega, Madison, WI); paraformaldehyde (PFA) (Labkemi, Stockholm, Sweden); BrdU Cell Proliferation ELISA (Roche Diagnostics Corporation, Indianapolis, IN); monoclonal *α*‐actinin antibody (Abcam, Cambridge, UK); polyclonal Ddr‐2 antibody (Santa Cruz, Santa Cruz, CA); Prolong gold antifade reagent (Invitrogen, Carlsbad, CA); Collagenase type 2 (Worthington, Lakewood, NJ).

### Buffers and media

The following buffers and media were used in the experiments: phosphate‐buffered saline pH 7.3 (PBS; 137 mmol/L NaCl, 27 mmol/L KCl, 6.74 mmol/L Na_2_HPO_4_x2H_2_0, 1.47 mmol/L KH_2_PO_4_ and 0.5% BSA**)**; PBS pH 7.3 (1.2% BSA); Tyrode's buffer pH 7.35 (140 mmol/L NaCl, 5 mmol/L KCl, 1 mmol/L MgCl_2_, 10 mmol/L Glucose, 10 mmol/L HEPES); starvation medium (DMEM, 1 mmol/L sodium pyruvate, 1% nonessential amino acids, 100 U/mL penicillin, and 100 *μ*g/mL streptomycin); complete medium (starvation medium with 10% fetal bovine serum); incubation buffer for measurement of cell size and multinucleation (0.1% saponin, 5% fetal bovine serum).

### Animal handling and cell culture

All animal procedures were in accordance with the local ethical committee on animal experiments (Linköping Ethical committee Dnr. 26‐10).

Fertilized Ross 308 fast growing broiler eggs were obtained from a local hatchery (Lantmännen Swehatch, Väderstad, Sweden). The eggs were kept at 18°C for no longer than 14 days before being incubated in commercial incubators (Model 25 HS, Masalles Comercial, Barcelona, Spain) set to 37.8°C, 45% humidity, and 21% O_2_. The eggs were turned automatically every hour.

Embryonic chicken cardiomyocytes (ECCMs) were obtained by enzymatic dissociation from hearts dissected from 19‐day‐old embryos euthanized by decapitation. The ventricles were dissected, cut into small pieces, and treated with an enzyme solution containing 160 U/mL collagenase and 0.78 U/mL protease for 15 min at 37°C followed by centrifugation for 5 min at 300 *g*. The resulting pellet was resuspended in Tyrode's buffer with 200 *μ*mol/L Ca^2+^ and put on ice. The procedure was repeated until all pieces were dissociated. The cell slurries were pooled, centrifuged, and resuspended in Tyrode's buffer with 400 *μ*mol/L Ca^2+^ followed by a second centrifugation. The pellet was resuspended in complete medium containing 1 mmol/L Ca^2+^ and put in the incubator for 1 h to preplate fibroblasts and endothelial cells. The cell suspension was centrifuged again and the pellet was resuspended in complete medium, seeded into cell culturing flasks, and incubated in a humidified atmosphere, 37°C and 5% CO_2_. Cardiomyocyte identity was confirmed by positive detection with an anti‐*α*‐actinin antibody as described below. Cardiomyocytes were kept in complete medium and split once a week (1:4).

### ECCM proliferation assay

Cells in passages 1–5 were used in all experiments. Preliminary experiments showed that 7000 cells per well on a 96‐well plate gave satisfactory levels for detecting both increase and decrease in proliferation (data not shown). Cells were seeded in medium containing 10% FBS. After 48 h of incubation, growth was arrested by changing to starvation medium for 24 h, after which the cells were incubated for another 24 h in medium supplemented with 0 or 2% FBS, in the absence (controls) or presence of IGF‐1 (1–100 ng/mL) or T_3_ (0.01–100 nmol/L).

Proliferation of ECCMs was analyzed using the CellTiter96^®^ Aqueous One Solution Cell Proliferation MTS Assay (Cory et al. 1991) and the BrdU incorporation assay, both according to the manufacturer's instructions. Proliferation was measured spectrophotometrically using a microplate reader (ASYS UVM 340, Biochrom, Camebridge, UK). All drugs and solvents used were tested for interference with the assays. In parallel, proliferation was also measured by manual cell counting in a Bürker chamber.

### Measurement of cell size and multinucleation

Embryonic chicken cardiomyocytes (3000/well) were seeded on chamber slides in medium containing 10% FBS. After 48‐h incubation, growth was arrested by changing to starvation medium for 24 h and incubated for another 24 h in medium supplemented with 0% FBS, in the absence (controls) or presence of IGF‐1 (10 ng/mL) or phenylephrine (10 and 100 *μ*mol/L) or in medium supplemented with 2% FBS in the absence (controls) or presence of T_3_ (10 and 100 nmol/L). The samples were washed twice in PBS (pH 7.3) and fixed for 30 min in 4% paraformaldehyde at RT. The samples were thereafter permeabilized and stained in a mixture of incubation buffer and bodipy phallacidin (0.6 *μ*g/mL) in PBS. The chamber slides were mounted with coverslips using mounting medium with DAPI nuclear stain and stored in +8°C prior to fluorescence microscopy analysis (Nikon Eclipse 80i, Tokyo, Japan). Area of the cells and number of nuclei were measured/counted using NIS Elements AR software (Nikon Instech Co Ltd, Tokyo, Japan).

### Characterization of cells

Cells obtained by enzymatic treatment of the chicken heart were characterized morphologically by fluorescent staining for *α*‐actinin (cardiomyocyte marker), F‐actin, Ddr‐2 (cardiac fibroblast marker), and *α*‐actin (smooth muscle cell marker). Cardiomyocytes were seeded in chamber slides at a density of 10,000 cells/well, incubated in complete medium for 48 h, and changed to starvation medium for 4 days. The samples were washed twice in PBS and fixed for 30 min in 4% paraformaldehyde at RT, permeabilized (0.5% Triton‐X), and stained for *α*‐actinin, Ddr‐2 or *α*‐actin (1:100 dilution of antibodies) for 1 h at RT. Incubation with Alexa Fluor 594 secondary antibody (1:400 dilution) and/or bodipy‐phallacidin (F‐actin) (0.6 *μ*g/mL) followed up for 1 h at RT and samples were subsequently mounted in fluorescent mounting medium and stored in +8°C prior to microscopy analysis. The slides were analyzed by confocal microscopy (Carl Zeiss, Oberkochen, Germany) or by fluorescence microscopy (Nikon Eclipse 80i). As a positive control of the antibodies, the Ddr‐2 and *α*‐actin antibodies were used to stain human fibroblasts from foreskin and human airway smooth muscle cells, respectively.

### Thyroid receptor mRNA expression

Embryonic chicken cardiomyocytes (500,000 cells per 35 mm Petri dish) were seeded in medium containing 10% FBS. Growth was arrested after 48‐h incubation by changing to starvation medium for 24 h and supplemented with 2% for another 24 h in the absence (controls) or presence of 10 nmol/L T_3_. Total RNA from 10^6^ cells was isolated using TRI reagent and reverse transcribed into cDNA using Revert Aid H Minus First strand cDNA synthesis kit with Oligo(dT)_18_ primers (Fermentas, Burlington, ON, Canada). Quantitative real‐time PCR was carried out using the Roche Light‐cycler 480 (Roche Applied Science, Roche Diagnostics, Basel, Switzerland) and Maxima SYBR Green qPCR master mix (Fermentas, Burlington, ON, Canada). Levels of *thyroid hormone receptor alpha (THRA)* transcript (NM_205313, forward primer: AAGCGCAAAAGAAAGAGCAGC, reverse primer: GTGATGCAGCGGTAGTGGTAG) were normalized to expression of *β*‐actin (NM_205518, forward primer: CACAGATCATGTTTGAGACCTT, reverse primer: CATCACAATACCAGTGGTACG) using the ΔCt‐method.

### Statistical analysis

Results are expressed as mean ± standard deviation. Gene expression data were analyzed using the 2^−ΔCt^‐values to express gene expression levels in the T_3_‐stimulated and control cells, respectively. Student's *t*‐test or one‐way ANOVA followed by Dunnet's multiple comparison test was used as statistical analyses. A *P*‐value < 0.05 was considered significant, and significance is denoted *(*P* < 0.05), **(*P* < 0.01) and ***(*P* < 0.001). Data were analyzed using GraphPad Prism^™^ version 5 (GraphPad Software, San Diego, CA).

## Results

**Figure 1.**
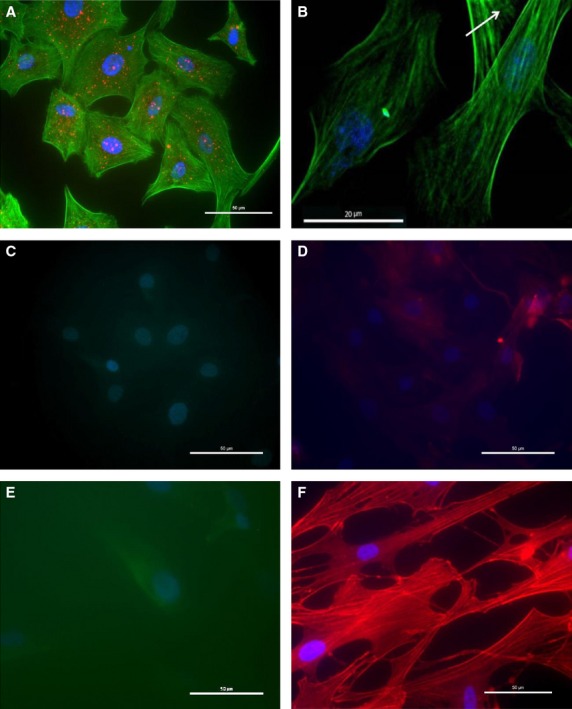
Cardiomyocyte identity (19‐day‐old embryonic chicken hearts (ECCMs)) was verified by immunocytochemistry using fluorescence (A C–F) or confocal microscopy (B). Positive identification for *α*‐actinin (red dots in A) and an incipient striation pattern (arrow in B) verifies the identity of the cells as cardiomyocytes. Fibroblasts and smooth muscle cells stain positive for fibroblast or smooth muscle‐specific markers, respectively, while cardiomyocytes do not. (A) ECCMs stained with bodipy phallacidin (green), monoclonal *α*‐actinin antibody (red) and DAPI (blue). (B) Bodipy phallacidin (green) and DAPI (blue). (C) polyclonal DDr‐2 antibody (green) and DAPI (blue). (D) monoclonal *α*‐actin antibody (red) and DAPI (blue). (E) Fibroblasts stained with polyclonal DDr‐2 antibody (green) and DAPI (blue). (F) smooth muscle cells stained with monoclonal *α*‐actin antibody (red) and DAPI (blue). The micrographs shown are representative from three independent experiments obtained from different cell passages. Calibration bar: 20 (B) or 50 (A, C–F) *μ*m.

We used cultured cardiomyocytes obtained by enzymatic dissociation from hearts dissected from 19‐day‐old chicken embryos in our experimental setup. Cardiomyocyte identity was confirmed using a primary cardiomyocyte‐specific anti‐*α*‐actinin antibody and an Alexa Fluor 594 secondary antibody, visualized in fluorescence/confocal microsope. The culture was found to consist only of cardiomyocytes, all cells stained positive for the cardiomyocyte‐specific structure *α*‐actinin (Fig. 1A) but not for Ddr‐2 (fibroblast specific, Fig. 1C) or *α*‐actin (smooth muscle cell specific, Fig. 1D). An incipient striation pattern was also observed in starved cells that stopped proliferating and started differentiating (Fig. 1B). The Ddr‐2 and *α*‐actin antibodies stained human fibroblasts from foreskin (Fig. 1E) and human airway smooth muscle cells (Fig 1F), respectively.

**Figure 2.**
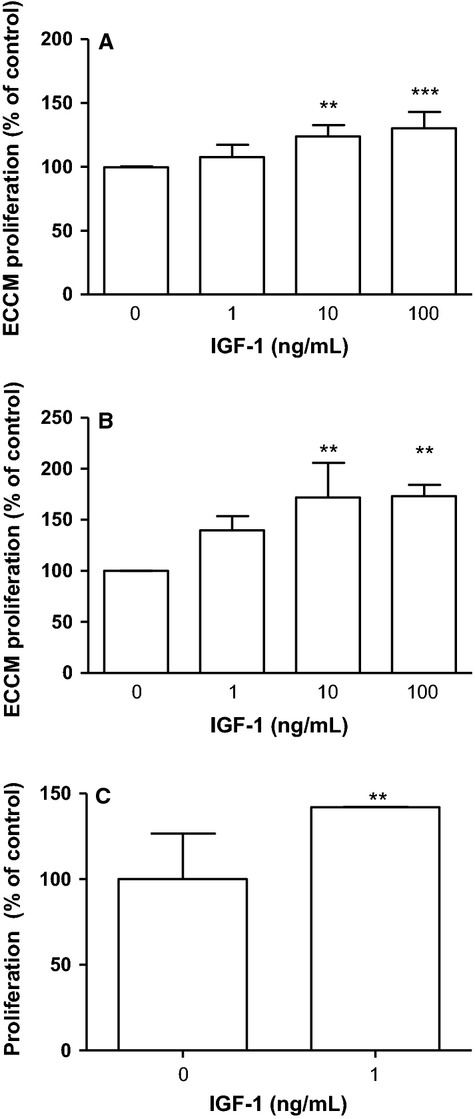
IGF‐1 stimulation for 24 h induce ECCM proliferation measured using the MTS‐assay (A), BrdU incorporation assay (B) and manual counting (C). Data are expressed as mean ± SD,* n* = 3–5 (A *n* = 5, B *n* = 4 and C *n* = 3) and One‐way ANOVA followed by Dunnet's post hoc was used for statistical analysis.

Stimulation with 10 *μ*mol/L IGF‐1 for 24 h triggered cell proliferation as shown by the 24% increase in the MTS assay (Fig. 2A) and a 72% increase in BrdU incorporation (Fig. 2B). In addition, stimulation with 1 *μ*mol/L IGF‐1 for 24 h resulted in a 42% significant increase in proliferation of ECCM measured using manual counting (Fig. 2C).

**Figure 3.**
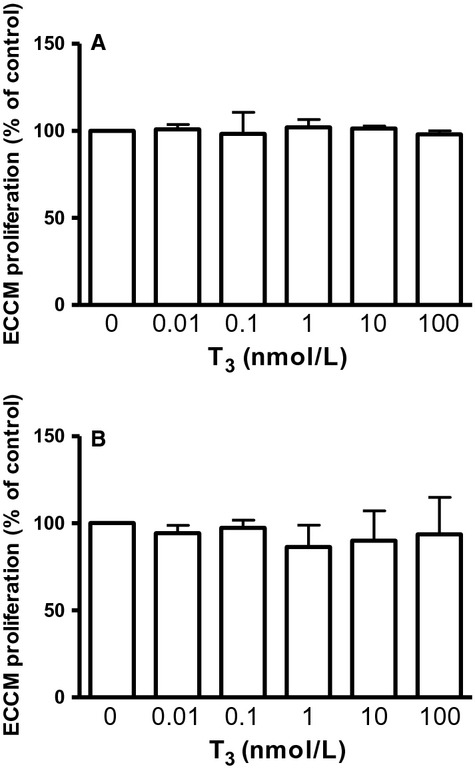
T_3_ stimulation for 24 h has no effect on ECCM proliferation using either the MTS (A) or the BrdU incorporation assays (B). Proliferation data expressed as mean ± SD (*n* = 13) and one‐way ANOVA followed by Dunnet's post hoc was used for statistical analysis.

**Figure 4.**
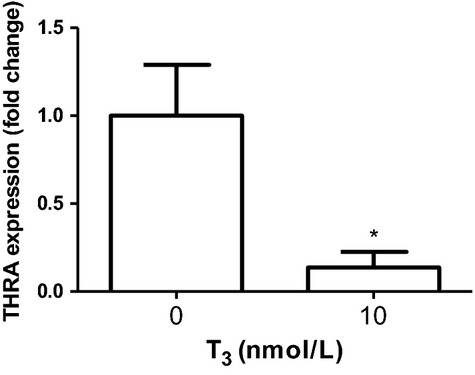
THRA mRNA expression in cultured cardiomyocytes subjected to T_3_ for 24 h was significantly decreased. Gene expression was normalized using *β*‐actin as the housekeeping gene and data are expressed as 2^−ΔCt^ normalized to control group level (*n* = 2). Student's *t*‐test was used for statistical analysis.

We also found that stimulation with T_3_ (in the presence of 2% FBS) did not have an effect on ECCM proliferation with either the MTS (Fig. 3A) or the BrdU incorporation assay (Fig. 3B) assay. ECCMs stimulated with 1 nmol/L T_3_ in the absence of FBS gave the same results (i.e. no effect on ECCM proliferation, ECCM: 100 ± 3.9%, ECCM + 1 nmol/L T3: 104 ± 2.8%, mean ± SD, *n* = 3). Quantitative real‐time PCR showed, however, that the chicken cardiomyocytes expressed the T_3_ receptor *α*‐isoform encoded by the THRA gene, and that stimulation with 10 nmol/L T_3_ induced an 86% significant downregulation in THRA expression (Fig. 4).

**Figure 5.**
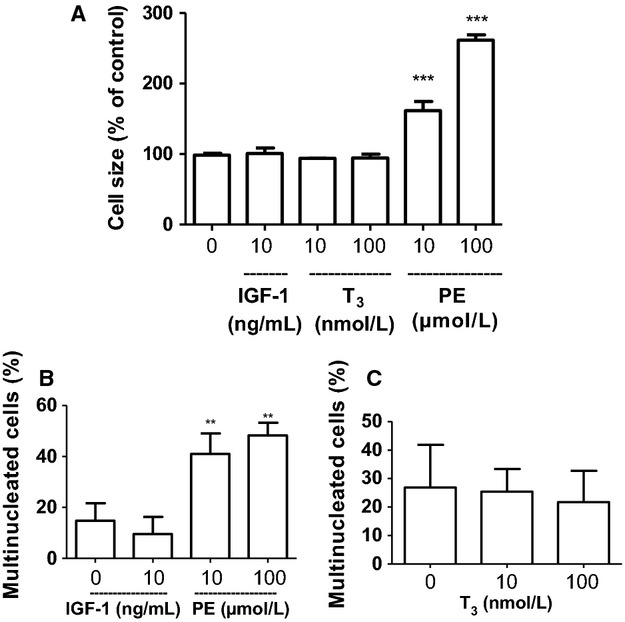
IGF‐1 or T_3_ stimulation for 24 h has no effect on ECCM hypertrophy (A) or multinucleation (B and C). Stimulation with phenylephrine (PE) for 24 h induced both hypertrophy and multinucleation (A and B). Stimulation with IGF‐1 or PE was done in the absence of FBS and stimulation with T_3_ was done in the presence of 2% FBS. The slides were studied by fluorescence microscopy (Nikon Eclipse 80i, Tokyo, Japan). Data are expressed as mean ± SD,* n* = 4–5 (A *n* = 4, B *n* = 5 and C *n* = 4) and one‐way ANOVA followed by Dunnet's post hoc was used for statistical analysis.

Phenylephrine (PE), a known hypertrophic stimulus, induced a significant increase in cell size (61% increase with 10 *μ*mol/L PE, Fig. 5A). The same dose of PE also induced cell multinucleation compared to controls (41% vs. 15%, respectively, Fig. 5B). However, neither T_3_ nor IGF had any effect on either cell size or multinucleation (Figs. 5A–C).

## Discussion

The growth trajectory of the heart and cardiomyocyte maturation during development differs greatly between species. It is compelling to look for correlations between heart maturation and the maturational stage of the entire organism at birth; alas, it is not that simple. While the heart cells are immature and still proliferating for another 1–3 weeks after birth in the altricial mouse and rat neonate (Clubb and Bishop 1984; Cluzeaut and Maurer‐Schultze 1986), cardiomyocytes in the precocial sheep and the altricial human are both fully differentiated at birth (Smolich et al. 1989; Barbera et al. 2000; Burrell et al. 2003; Thornburg et al. 2011). The precocial chicken, however, has 100% mononucleated, proliferating cardiomyocytes at hatching and 44% of that cell population is maintained in a proliferative state at 6 weeks of age (Li et al. 1997). Many physiologically occurring factors that suppress cardiomyocyte proliferation have been identified in mammalian models, out of which T_3_ seems to be one of the most potent drivers of cardiomyocyte maturation (Kinugawa et al. 2005; Chattergoon et al. 2012a). As the HPA–thyroid axis develops in the second half of gestation in sheep and human (Thorpe‐Beeston et al. 1991; Polk 1995; Fisher et al. 2000), T_3_ increases exponentially and results in a prepartum surge of the hormone (Thornburg et al. 2011). Despite the fact that a similar ontogeny of HPA axis function is present in the chicken, including the prepartum/prehatching surge of T_3_, in vivo chicken cardiomyocyte proliferation seems to be unaffected by the hormone since the cells keep dividing far beyond hatching. By studying how embryonic chicken cardiomyocytes react to controlled exposure to T_3_, we show that the cells are insensitive to T_3_ stimulation in vitro and that the hormone has no effect on cell size or maturation shown by cell binucleation.

### IGF‐1 increases cardiomyocyte proliferation

Insulin‐like growth factor‐1 is well documented as a pro‐proliferative factor in a range of organisms and cell types (Kardami 1990; Resnicoff et al. 1993; Cao et al. 2003; Sundgren et al. 2003b). After IGF‐1 stimulates either the IGF‐1 or insulin receptor, the intracellular signal is transmitted through the PI3K/Akt and/or MAPK pathways resulting in increased proliferation (Sundgren et al. 2003b). Interestingly, MAPK and PI3K/Akt signaling pathways are involved in both hypertrophic responses in the mature heart as well as regulation of proliferation before cardiomyocyte maturation. Functional MAPK signaling (through the ERK limb) and PI3K signaling are both required for IGF‐1 and angiotensin II (Ang‐II)‐induced hyperplasia (Sundgren et al. 2003a,b). Functional MAPK signaling is also required for Ang‐II‐ and TGF*β*‐induced hypertrophic responses in mature cardiomyocytes (Schultz et al. 2002; Watkins et al. 2011). As expected, the proliferative action of IGF‐1 is also present in embryonic chicken cardiomyocytes and has no effect on binucleation or cell size. The involvement of the PI3K and MAPK pathway in the chicken cardiomyocyte response to IGF‐1 is not known and an interesting area for future research.

### Thyroid hormone receptor α expression is decreased with T_3_ stimulation

The concentration of circulating T_3_ in the mammalian fetus rises in late gestation as the fetal hypothalamic–pituitary–thyroid axis matures (White et al. 2001). T_3_ in late development is important for the maturation of different fetal tissues and fetal hypothyroidism that affects growth (Latimer et al. 1993; Fowden 1995; Fowden and Silver 1995; Forhead et al. 1998). To keep bioavailable thyroid hormone at a constant level, T_3_ is involved in a series of autoregulatory mechanisms. Thyroid‐stimulating hormone (TSH) and thyrotropin‐releasing hormone (TRH) are both negatively controlled by T_3_ (Gauthier et al. 1999), leading to a negative feedback loop when T_3_ levels increase and thus decreasing hormone release. Regulation of the thyroid axis also takes place on the transcriptional level of the thyroid receptors. T_3_ mediates its effects through binding to nuclear hormone receptors (*α*,* β*‐1 and *β*‐2) and activating intracellular signaling pathways dependent on mitogen‐activated protein kinase p38 (Kinugawa et al. 2005). The receptor type THRA ontologically precedes the THRB and is the predominantly expressed receptor type during development in several species studied (Lazar 1993). The expression of THRA decreases and THRB increases closer to term, possibly induced by cortisol (Forhead and Fowden 2014). Cortisol upregulates the deiodinases converting T_4_ to T_3_ and thereby increasing the bioavailability of T_3_ (Chattergoon et al. 2012a). While THRB expression is negatively affected by TRH, THRA is directly influenced by T_3_ and mRNA levels of THRA has been shown to decrease with increased T_3_ concentration (Lazar 1993). We verified the presence of THRA expression in the developing chicken heart and observed a decreased expression with thyroid stimulation indicative of an active T_3_ signaling cascade. We believe that this change in receptor gene expression excludes the possibility that the absence of T_3_ effects is due to the inability of chicken cardiomyocytes to continue proliferating in culture as previously shown (Clark and Fischman 1983).

### Embryonic chicken cardiomyocyte proliferation or maturation is not affected by T_3_

T_3_ mediates decreased hyperplasia and/or maturation and hypertrophy through activation of intracellular signaling pathways dependent on p38 (Kinugawa et al. 2005), p21, cyclin D1 (Chattergoon et al. 2012a,b), phospho‐mTOR, ANP, and SERCA2a (Chattergoon et al. 2012a). Among them, cyclin D1 and p21 are key proteins of the cell cycle. Cyclin D1 associates with the catalytic kinase units CDK4 and CDK6 to sequester the CDK‐inhibitory proteins p21 and p27 to relieve the inhibition of CDK2. This allows the cell to enter the S phase and thereby proliferate. A suppression of cyclin D1 or an increase of p21 can therefore potentially decrease cell proliferation. In fetal sheep cardiomyocytes, T_3_ does both (Chattergoon et al. 2012a). Although the MAPK/ERK pathway is implied in the proliferative mechanism as described above, the increase in p21 also occurs through the well‐established hypertrophic ERK/mTOR pathway (Chattergoon et al. 2012a). The mechanism through which ERK can mediate both proliferation and hypertrophy is not fully understood, but has been connected to transient vs. sustained ERK activation (Bottazzi et al. 1999). The mature chicken cardiomyocyte is less differentiated and more primitive than the mammalian. For example, the shape is more spindle‐like compared to the brick‐like mammalian and chicken cardiomyocytes additionally lack *t*‐tubulus (Sommer and Johnson 1968). Thus, it is not too farfetched to imagine that chicken cardiomyocytes also display differences in the molecular pathways controlling proliferation and growth. It has also been postulated that the reason behind that such exclusive processes as proliferation and hypertrophy can be mediated through the MAPK/ERK pathway may be through a “remodeling” of the signaling cascade with maturation (Sundgren et al. 2003a), possibly through microdomain alterations when the physical cardiomyocyte structure becomes more complex. If this is the case, the chicken cardiomyocyte with its comparatively low complexity may never go through the “remodeling” of a more differentiated cardiomyocyte and maintain a more primitive signaling phenotype. Future studies should be aimed at dissecting out the role of ERK in such different cell responses as proliferation and hypertrophy.

## Acknowledgments

We acknowledge the help of Huma Syeda Zahra Naqvi for the help with cell size measurements and Shehla Irrum for her pilot work on chicken cardiomyocyte isolation.

## Conflict of Interest

None declared.
